# Diterbium hepta­nickel: a crystal structure redetermination

**DOI:** 10.1107/S1600536814015384

**Published:** 2014-07-05

**Authors:** Volodymyr Levytskyy, Volodymyr Babizhetskyy, Bohdan Kotur, Volodymyr Smetana

**Affiliations:** a Department of Inorganic Chemistry, Ivan Franko National University of Lviv, Kyryla & Mefodiya street 6, 79005 Lviv, Ukraine; b 344 Spedding Hall, Ames Laboratory, Ames, IA 50011-3020, USA

**Keywords:** crystal structure

## Abstract

The crystal structure of the title compound, Tb_2_Ni_7_, was redetermined from single-crystal X-ray diffraction data. In comparison with previous studies based on powder X-ray diffraction data [Lemaire *et al.* (1967). *C. R. Acad. Sci. Ser. B*, **265**, 1280–1282; Lemaire & Paccard (1969). *Bull. Soc. Fr. Mineral. Cristallogr.* **92**, 9–16; Buschow & van der Goot (1970). *J. Less-Common Met.* **22**, 419–428], the present redetermination affords refined coordinates and anisotropic displacement parameters for all atoms. A partial occupation for one Tb atom results in the non-stoichiometric composition Tb_1.962 (4)_Ni_7_. The title compound adopts the Ce_2_Ni_7_ structure type and can also be derived from the CaCu_5_ structure type as an inter­growth structure. The asymmetric unit contains two Tb sites (both site symmetries 3*m*.) and five Ni sites (.*m*., *mm*2, 3*m*., 3*m*., -3*m*.). The two different coordination polyhedra of Tb are a Frank–Kasper polyhedron formed by four Tb and 12 Ni atoms and a pseudo Frank–Kasper polyhedron formed by two Tb and 18 Ni atoms. The four different coordination polyhedra of Ni are Frank–Kasper icosa­hedra formed by five Tb and seven Ni atoms, four Tb and eight Ni atoms, three Tb and nine Ni atoms, and six Tb and six Ni atoms, respectively.

## Related literature   

For the Ce_2_Ni_7_ structure type, see: Cromer & Larson (1959 ▶). For previous X-ray powder studies of the title compound, see: Lemaire *et al.* (1967 ▶); Lemaire & Paccard (1969 ▶); Buschow & van der Goot (1970 ▶). For related compounds, see: Bertaut *et al.* (1965 ▶); Virkar & Raman (1969 ▶); Buschow & van der Goot (1970 ▶); Paul-Boncour *et al.* (2006 ▶); Levytskyy *et al.* (2012 ▶). For inter­growth structures, see: Parthé *et al.* (1985 ▶); Grin (1992 ▶). For standardization of crystal structure data, see: Gelato & Parthé (1987 ▶).

## Experimental   

### 

#### Crystal data  

Tb_1.96_Ni_7_
*M* *_r_* = 722.72
Hexagonal, 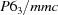

*a* = 4.944 (1) Å
*c* = 24.129 (6) Å
*V* = 510.8 (2) Å^3^
*Z* = 4
Mo *K*α radiation
μ = 51.78 mm^−1^
*T* = 293 K
0.05 × 0.04 × 0.04 mm

#### Data collection  

Bruker SMART CCD diffractometer
Absorption correction: multi-scan (*SADABS*; Bruker, 2007 ▶) *T*_min_ = 0.50, *T*_max_ = 0.74
7652 measured reflections
422 independent reflections
313 reflections with *I* > 2σ(*I*)
*R*_int_ = 0.073

#### Refinement  

*R*[*F* ^2^ > 2σ(*F* ^2^)] = 0.025
*wR*(*F* ^2^) = 0.041
*S* = 1.07
422 reflections
27 parameters
Δρ_max_ = 1.51 e Å^−3^
Δρ_min_ = −1.76 e Å^−3^

### 

Data collection: *SMART* (Bruker, 2007 ▶); cell refinement: *SAINT* (Bruker, 2007 ▶); data reduction: *SAINT*; program(s) used to solve structure: *SIR2011* (Burla *et al.*, 2012 ▶); program(s) used to refine structure: *SHELXL2013* (Sheldrick, 2008 ▶) and *WinGX* (Farrugia, 2012 ▶); molecular graphics: *DIAMOND* (Brandenburg, 2006 ▶); software used to prepare material for publication: *publCIF* (Westrip, 2010 ▶).

## Supplementary Material

Crystal structure: contains datablock(s) I. DOI: 10.1107/S1600536814015384/fj2679sup1.cif

Structure factors: contains datablock(s) I. DOI: 10.1107/S1600536814015384/fj2679Isup2.hkl

CCDC reference: 1011470

Additional supporting information:  crystallographic information; 3D view; checkCIF report

## Figures and Tables

**Fig. 1.**
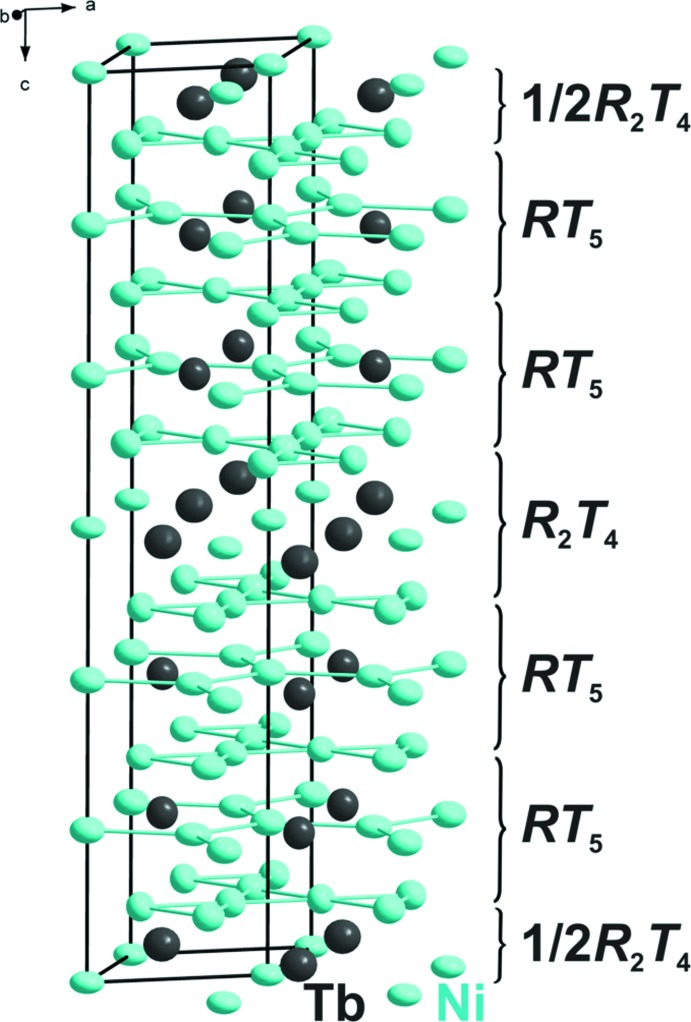
Perspective view of the crystal structure of Tb2Ni7. The unit cell and the blocks of RT5 and R2T4 are emphasized. Atoms are represented by their anisotropic displacement ellipsoids at the 99.99% probability level

**Fig. 2.**
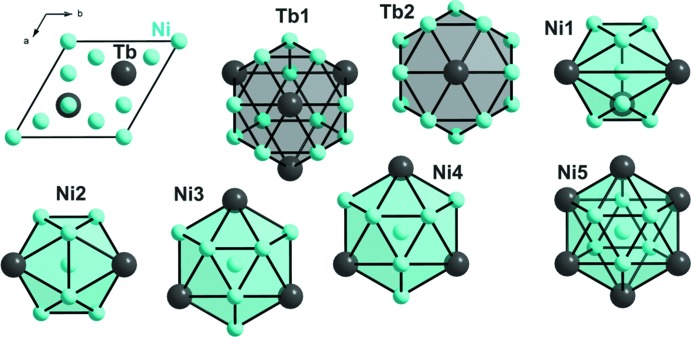
The ab projection of the unit cell and coordination polyhedra for all types of atoms in the Tb2Ni7 structure

## supplementary crystallographic information

### S1. Comment

A lot of works have been published about *R*_2_Ni_7_ stoichiometry compounds (*R* = rare earth element) (see, Lemaire *et al.*, 1967; Lemaire & Paccard, 1969; Virkar & Raman, 1969; Buschow & van der Goot, 1970) with either β-Gd_2_Co_7_ (Bertaut *et al.*, 1965) or Ce_2_Ni_7_ (Cromer & Larson, 1959) structure types. According to Virkar & Raman (1969) the high-temperature modifications adopt the rhombohedral β-Gd_2_Co_7_ type structure whereas the low-temperature phases are isomorphic with Ce_2_Ni_7_. On the other hand, Lemaire *et al.* (1967) observed the coexistence of both modifications even in annealed at 1373 K samples for Pr_2_Ni_7_, Nd_2_Ni_7_, Gd_2_Ni_7_, Tb_2_Ni_7_, and Dy_2_Ni_7_. Buschow & van der Goot (1970) investigated series of *R*_2_Ni_7_ compounds and concluded the crystal structures of the *R*_2_Ni_7_ compounds are dependent on the *R* atom size. The transformation between these two polymorphic forms is of a martensitic type.

Our research work mainly deals with the heavy rare earth – transition metal (*R*–*T*) systems. And the crystal structures of the compounds forming in such systems are of the most interest. Investigation of the Tb–Ni system at 1070 K resulted in good agreement with the literature data for unit cell parameters for all compounds obtained from powder X-ray diffraction using starting coordinates of appropriate structure types. It was noted there is no any information in literature about crystal structure refinement of heavy rare earth *R*_2_Ni_7_ compounds. Our recent work was devoted to the refinement of the Dy_2_Ni_7_ compound, which crystal stucture is isomorphous with β-Gd_2_Co_7_ (see, Levytskyy *et al.*, 2012). In present study the crystal structure of Tb_2_Ni_7_ was redetermined with high accurracy using single-crystal X-ray method.

The structure is characterized by two independent terbium atom sites (both 4*f* Wyckoff positions) and five nickel atom sites (12*k*, 6*h*, 4*f*, 4*e* and 2*a*). The unit cell of diterbium heptanickel is shown in Fig. 1. The structure may be viewed as staking of *RT*_5_ blocks corresponding to the CaCu_5_-type and *R*_2_*T*_4_ blocks corresponding to the MgCu_2_-type structures. The presence of the same Kagome net in the structure types of CaCu_5_ and the Laves phase MgCu_2_ allows a combination of both structural motifs along the 6_3_ screw axis giving an intergrowth structure: 4*RT*_5_ + 2*R*_2_*T*_4_ = 4*R*_2_*T*_7_ (Parthé *et al.*, 1985; Grin, 1992).

In Fig. 2 the *ab* projection of the unit cell and the coordination polyhedra for all atom types are shown. The coordination number for all Ni atoms is 12. The coordination polyhedra are Frank–Kasper icosahedra. The Ni1 atom (Wyckoff site 12*k*, site symmetry. *m*.) is surrounded by 5 Tb atoms and 7 Ni atoms. The Ni2 atom (Wyckoff site 6*h*, site symmetry *mm*2) is surrounded by 4 Tb atoms and 8 Ni atoms. The Ni3 and Ni4 atoms (Wyckof sites 4*f* and 4*e*, site symmetries 3*m*.) are surrounded by 3 Tb atoms and 9 Ni atoms. The Ni5 (Wyckoff site 2*a*, site symmetry 3*m*.) is surrounded by 6 Tb and 6 Ni atoms. The coordination polyhedra for Tb1 and Tb2 atoms (Wyckoff sites 4*f*, site symmetries 3*m*.) are a Frank–Kasper polyhedron (coordination number 16) and a pseudo Frank–Kasper polyhedron (coordination number 20), respectively. The Tb1 atom is surrounded by 4 Tb atoms and 12 Ni atoms. The Tb2 atom is surrounded by 2 Tb atoms and 18 Ni atoms.

### S2. Experimental

The sample was prepared from powdered commercially available pure elements: sublimed bulk pieces of terbium metal with a claimed purity of 99.9 at.% (Strem Chemicals) and 99.99% pure nickel powder (Aldrich Chem. Inc.). A mixture of the powders was compacted into a pellet. It was arc-melted under an argon atmosphere on a water-cooled copper hearth. The alloy button (~1 g) was turned over and remelted three times to improve homogeneity. Subsequently, the sample was annealed in an evacuated silica tube for four weeks at 1070 K. Shiny metallic gray prysmatic shaped crystals were isolated mechanically from crushed sample with a help of microscope.

### S3. Refinement

The atomic positions found from the direct methods structure solution were in good agreement with those from the Ce_2_Ni_7_ structure type (Cromer & Larson, 1959) and were used as starting point for the structure refinement. An increased value of isotropic thermal parameter for Tb1 atom was observed. Refined occupation of the site is 96.2 (4)% resulting in composition Tb_1.962 (4)_Ni_7_. Interatomic distances Tb1–Tb1 (in *R*_2_*T*_4_ blocks) are slightly decreased (3.173 (1) Å) and correlate with those observed in Tb_1 -_*_x_*Ni_2_ (3.11 Å) (Paul-Boncour *et al.*, 2006). Atomic positions were standardized using program *STRUCTURE TIDY* (Gelato & Parthé, 1987). The highest Fourier difference peak of 1.51 e·Å^-3^ is at (1/3 2/3 0.041) and 1.68 Å away from Tb1 atom. The deepest hole (-1.76 e·Å^-3^) is at (2/3 1/3 0.199) and 0.62 Å away from Tb2 atom.

### Crystal data

**Table:**

Tb_1.96_Ni_7_	*D*_x_ = 9.398 Mg m^−^^3^
*M**_r_* = 722.72	Mo *K*α radiation, λ = 0.71073 Å
Hexagonal, *P*6_3_/*m**m**c*	Cell parameters from 7652 reflections
*a* = 4.944 (1) Å	θ = 1.7–32.8°
*c* = 24.129 (6) Å	µ = 51.78 mm^−^^1^
*V* = 510.8 (2) Å^3^	*T* = 293 K
*Z* = 4	Irregular, fragment, metallic gray
*F*(000) = 1294	0.05 × 0.04 × 0.04 mm

### Data collection

**Table:**

Bruker SMART CCD diffractometer	313 reflections with *I* > 2σ(*I*)
Radiation source: fine-focus sealed tube	*R*_int_ = 0.073
ω scans	θ_max_ = 32.8°, θ_min_ = 1.7°
Absorption correction: multi-scan (*SADABS*; Bruker, 2007)	*h* = −7→7
*T*_min_ = 0.50, *T*_max_ = 0.74	*k* = −7→7
7652 measured reflections	*l* = −36→35
422 independent reflections	

### Refinement

**Table:**

Refinement on *F*^2^	0 restraints
Least-squares matrix: full	*w* = 1/[σ^2^(*F*_o_^2^) + (0.0109*P*)^2^ + 2.7514*P*] where *P* = (*F*_o_^2^ + 2*F*_c_^2^)/3
*R*[*F*^2^ > 2σ(*F*^2^)] = 0.025	(Δ/σ)_max_ = 0.001
*wR*(*F*^2^) = 0.041	Δρ_max_ = 1.51 e Å^−^^3^
*S* = 1.07	Δρ_min_ = −1.76 e Å^−^^3^
422 reflections	Extinction correction: *SHELXL2013* (Sheldrick, 2008), Fc^*^=kFc[1+0.001xFc^2^λ^3^/sin(2θ)]^-1/4^
27 parameters	Extinction coefficient: 0.00061 (6)

### Special details

**Table:**

Geometry. All e.s.d.'s (except the e.s.d. in the dihedral angle between two l.s. planes) are estimated using the full covariance matrix. The cell e.s.d.'s are taken into account individually in the estimation of e.s.d.'s in distances, angles and torsion angles; correlations between e.s.d.'s in cell parameters are only used when they are defined by crystal symmetry. An approximate (isotropic) treatment of cell e.s.d.'s is used for estimating e.s.d.'s involving l.s. planes.

### Fractional atomic coordinates and isotropic or equivalent isotropic displacement parameters (Å^2^)

**Table:**

	*x*	*y*	*z*	*U*_iso_*/*U*_eq_	Occ. (<1)
Ni1	0.16717 (13)	0.3343 (3)	0.08560 (3)	0.0076 (2)	
Ni2	0.1665 (2)	0.3330 (4)	0.2500	0.0076 (3)	
Ni3	0.3333	0.6667	0.16728 (6)	0.0088 (3)	
Tb1	0.3333	0.6667	0.52871 (2)	0.0108 (2)	0.962 (4)
Tb2	0.3333	0.6667	0.67352 (2)	0.00849 (16)	
Ni4	0.0000	0.0000	0.16750 (6)	0.0089 (3)	
Ni5	0.0000	0.0000	0.0000	0.0081 (5)	

### Atomic displacement parameters (Å^2^)

**Table:**

	*U*^11^	*U*^22^	*U*^33^	*U*^12^	*U*^13^	*U*^23^
Ni1	0.0082 (4)	0.0065 (4)	0.0073 (4)	0.0033 (2)	−0.0001 (2)	−0.0002 (4)
Ni2	0.0096 (5)	0.0067 (6)	0.0055 (5)	0.0033 (3)	0.000	0.000
Ni3	0.0111 (5)	0.0111 (5)	0.0044 (7)	0.0055 (2)	0.000	0.000
Tb1	0.0114 (3)	0.0114 (3)	0.0098 (3)	0.00568 (13)	0.000	0.000
Tb2	0.0083 (2)	0.0083 (2)	0.0090 (3)	0.00413 (10)	0.000	0.000
Ni4	0.0103 (5)	0.0103 (5)	0.0060 (7)	0.0052 (3)	0.000	0.000
Ni5	0.0100 (8)	0.0100 (8)	0.0043 (9)	0.0050 (4)	0.000	0.000

### Geometric parameters (Å, º)

**Table:**

Ni1—Ni3	2.4309 (15)	Tb1—Ni1^xviii^	2.8274 (7)
Ni1—Ni4	2.4403 (15)	Tb1—Ni5^xv^	2.9373 (6)
Ni1—Ni1^i^	2.465 (2)	Tb1—Ni5^xix^	2.9373 (6)
Ni1—Ni1^ii^	2.465 (2)	Tb1—Ni5^xvii^	2.9373 (6)
Ni1—Ni1^iii^	2.479 (2)	Tb1—Ni1^xx^	3.1036 (12)
Ni1—Ni1^iv^	2.479 (2)	Tb1—Ni1^vii^	3.1036 (12)
Ni1—Ni5	2.5129 (10)	Tb1—Ni1^xxi^	3.1036 (12)
Ni1—Tb1^v^	2.8274 (7)	Tb2—Ni4^xv^	2.8581 (6)
Ni1—Tb1^vi^	2.8275 (7)	Tb2—Ni4^xix^	2.8581 (6)
Ni1—Tb1^vii^	3.1036 (12)	Tb2—Ni4^xvii^	2.8581 (6)
Ni1—Tb2^v^	3.2576 (8)	Tb2—Ni3^xvii^	2.8584 (6)
Ni1—Tb2^vi^	3.2576 (8)	Tb2—Ni3^xv^	2.8584 (6)
Ni2—Ni4^vii^	2.4485 (16)	Tb2—Ni3^xxii^	2.8584 (6)
Ni2—Ni4	2.4486 (16)	Tb2—Ni2^xxiii^	3.0848 (6)
Ni2—Ni3^vii^	2.4545 (16)	Tb2—Ni2^xxiv^	3.0848 (6)
Ni2—Ni3	2.4545 (16)	Tb2—Ni2^viii^	3.0848 (6)
Ni2—Ni2^iii^	2.470 (3)	Tb2—Ni2^xxv^	3.0849 (6)
Ni2—Ni2^iv^	2.470 (3)	Tb2—Ni2^ix^	3.0849 (6)
Ni2—Ni2^i^	2.474 (3)	Tb2—Ni2^xxvi^	3.0849 (6)
Ni2—Ni2^ii^	2.474 (3)	Ni4—Ni1^iii^	2.4403 (15)
Ni2—Tb2^viii^	3.0848 (6)	Ni4—Ni1^iv^	2.4403 (15)
Ni2—Tb2^v^	3.0848 (6)	Ni4—Ni2^iv^	2.4486 (16)
Ni2—Tb2^vi^	3.0848 (6)	Ni4—Ni2^iii^	2.4486 (16)
Ni2—Tb2^ix^	3.0848 (6)	Ni4—Ni3^xxvii^	2.8544 (6)
Ni3—Ni1^i^	2.4310 (15)	Ni4—Ni3^xxviii^	2.8544 (6)
Ni3—Ni1^ii^	2.4310 (15)	Ni4—Tb2^v^	2.8581 (6)
Ni3—Ni2^i^	2.4545 (16)	Ni4—Tb2^xxix^	2.8581 (6)
Ni3—Ni2^ii^	2.4545 (16)	Ni4—Tb2^vi^	2.8581 (6)
Ni3—Ni4^x^	2.8544 (6)	Ni5—Ni1^xxx^	2.5129 (10)
Ni3—Ni4^xi^	2.8544 (6)	Ni5—Ni1^xxxi^	2.5129 (10)
Ni3—Ni4	2.8544 (6)	Ni5—Ni1^iv^	2.5129 (10)
Ni3—Tb2^v^	2.8584 (6)	Ni5—Ni1^iii^	2.5129 (10)
Ni3—Tb2^xii^	2.8584 (6)	Ni5—Ni1^xxxii^	2.5129 (10)
Ni3—Tb2^vi^	2.8584 (6)	Ni5—Tb1^xxxiii^	2.9373 (6)
Tb1—Ni1^xiii^	2.8274 (7)	Ni5—Tb1^v^	2.9373 (6)
Tb1—Ni1^xiv^	2.8274 (7)	Ni5—Tb1^vii^	2.9373 (6)
Tb1—Ni1^xv^	2.8274 (7)	Ni5—Tb1^xxix^	2.9373 (6)
Tb1—Ni1^xvi^	2.8274 (7)	Ni5—Tb1^vi^	2.9373 (6)
Tb1—Ni1^xvii^	2.8274 (7)	Ni5—Tb1^xxxiv^	2.9373 (6)
			
Ni3—Ni1—Ni4	71.74 (4)	Ni1^xviii^—Tb1—Ni5^xvii^	96.49 (2)
Ni3—Ni1—Ni1^i^	59.54 (3)	Ni5^xv^—Tb1—Ni5^xvii^	114.616 (9)
Ni4—Ni1—Ni1^i^	120.53 (3)	Ni5^xix^—Tb1—Ni5^xvii^	114.617 (9)
Ni3—Ni1—Ni1^ii^	59.54 (3)	Ni1^xiii^—Tb1—Ni1^xx^	115.511 (19)
Ni4—Ni1—Ni1^ii^	120.53 (3)	Ni1^xiv^—Tb1—Ni1^xx^	94.85 (3)
Ni1^i^—Ni1—Ni1^ii^	60.0	Ni1^xv^—Tb1—Ni1^xx^	94.85 (3)
Ni3—Ni1—Ni1^iii^	120.46 (3)	Ni1^xvi^—Tb1—Ni1^xx^	115.511 (19)
Ni4—Ni1—Ni1^iii^	59.47 (3)	Ni1^xvii^—Tb1—Ni1^xx^	141.157 (16)
Ni1^i^—Ni1—Ni1^iii^	120.0	Ni1^xviii^—Tb1—Ni1^xx^	141.157 (16)
Ni1^ii^—Ni1—Ni1^iii^	180.0	Ni5^xv^—Tb1—Ni1^xx^	49.07 (2)
Ni3—Ni1—Ni1^iv^	120.46 (3)	Ni5^xix^—Tb1—Ni1^xx^	90.753 (19)
Ni4—Ni1—Ni1^iv^	59.47 (3)	Ni5^xvii^—Tb1—Ni1^xx^	90.753 (19)
Ni1^i^—Ni1—Ni1^iv^	180.0	Ni1^xiii^—Tb1—Ni1^vii^	141.157 (16)
Ni1^ii^—Ni1—Ni1^iv^	120.0	Ni1^xiv^—Tb1—Ni1^vii^	141.157 (16)
Ni1^iii^—Ni1—Ni1^iv^	60.0	Ni1^xv^—Tb1—Ni1^vii^	115.511 (19)
Ni3—Ni1—Ni5	178.90 (5)	Ni1^xvi^—Tb1—Ni1^vii^	94.85 (3)
Ni4—Ni1—Ni5	109.36 (5)	Ni1^xvii^—Tb1—Ni1^vii^	115.511 (19)
Ni1^i^—Ni1—Ni5	119.56 (2)	Ni1^xviii^—Tb1—Ni1^vii^	94.85 (3)
Ni1^ii^—Ni1—Ni5	119.56 (2)	Ni5^xv^—Tb1—Ni1^vii^	90.753 (18)
Ni1^iii^—Ni1—Ni5	60.44 (2)	Ni5^xix^—Tb1—Ni1^vii^	49.07 (2)
Ni1^iv^—Ni1—Ni5	60.44 (2)	Ni5^xvii^—Tb1—Ni1^vii^	90.753 (19)
Ni3—Ni1—Tb1^v^	113.23 (3)	Ni1^xx^—Tb1—Ni1^vii^	46.79 (4)
Ni4—Ni1—Tb1^v^	113.09 (3)	Ni1^xiii^—Tb1—Ni1^xxi^	94.85 (3)
Ni1^i^—Ni1—Tb1^v^	116.01 (2)	Ni1^xiv^—Tb1—Ni1^xxi^	115.511 (19)
Ni1^ii^—Ni1—Tb1^v^	64.16 (2)	Ni1^xv^—Tb1—Ni1^xxi^	141.157 (16)
Ni1^iii^—Ni1—Tb1^v^	115.84 (2)	Ni1^xvi^—Tb1—Ni1^xxi^	141.157 (16)
Ni1^iv^—Ni1—Tb1^v^	63.99 (2)	Ni1^xvii^—Tb1—Ni1^xxi^	94.85 (3)
Ni5—Ni1—Tb1^v^	66.43 (2)	Ni1^xviii^—Tb1—Ni1^xxi^	115.511 (19)
Ni3—Ni1—Tb1^vi^	113.23 (3)	Ni5^xv^—Tb1—Ni1^xxi^	90.753 (19)
Ni4—Ni1—Tb1^vi^	113.09 (3)	Ni5^xix^—Tb1—Ni1^xxi^	90.753 (18)
Ni1^i^—Ni1—Tb1^vi^	64.16 (2)	Ni5^xvii^—Tb1—Ni1^xxi^	49.07 (2)
Ni1^ii^—Ni1—Tb1^vi^	116.01 (2)	Ni1^xx^—Tb1—Ni1^xxi^	46.79 (4)
Ni1^iii^—Ni1—Tb1^vi^	63.99 (2)	Ni1^vii^—Tb1—Ni1^xxi^	46.79 (4)
Ni1^iv^—Ni1—Tb1^vi^	115.84 (2)	Ni4^xv^—Tb2—Ni4^xix^	119.744 (6)
Ni5—Ni1—Tb1^vi^	66.43 (2)	Ni4^xv^—Tb2—Ni4^xvii^	119.744 (6)
Tb1^v^—Ni1—Tb1^vi^	121.92 (4)	Ni4^xix^—Tb2—Ni4^xvii^	119.744 (6)
Ni3—Ni1—Tb1^vii^	116.89 (5)	Ni4^xv^—Tb2—Ni3^xvii^	174.07 (5)
Ni4—Ni1—Tb1^vii^	171.37 (5)	Ni4^xix^—Tb2—Ni3^xvii^	59.912 (1)
Ni1^i^—Ni1—Tb1^vii^	66.606 (18)	Ni4^xvii^—Tb2—Ni3^xvii^	59.912 (1)
Ni1^ii^—Ni1—Tb1^vii^	66.606 (18)	Ni4^xv^—Tb2—Ni3^xv^	59.911 (2)
Ni1^iii^—Ni1—Tb1^vii^	113.392 (18)	Ni4^xix^—Tb2—Ni3^xv^	59.912 (2)
Ni1^iv^—Ni1—Tb1^vii^	113.392 (18)	Ni4^xvii^—Tb2—Ni3^xv^	174.07 (5)
Ni5—Ni1—Tb1^vii^	62.01 (2)	Ni3^xvii^—Tb2—Ni3^xv^	119.726 (6)
Tb1^v^—Ni1—Tb1^vii^	64.488 (19)	Ni4^xv^—Tb2—Ni3^xxii^	59.911 (2)
Tb1^vi^—Ni1—Tb1^vii^	64.490 (19)	Ni4^xix^—Tb2—Ni3^xxii^	174.07 (5)
Ni3—Ni1—Tb2^v^	58.178 (19)	Ni4^xvii^—Tb2—Ni3^xxii^	59.912 (1)
Ni4—Ni1—Tb2^v^	58.115 (19)	Ni3^xvii^—Tb2—Ni3^xxii^	119.726 (6)
Ni1^i^—Ni1—Tb2^v^	112.369 (19)	Ni3^xv^—Tb2—Ni3^xxii^	119.725 (6)
Ni1^ii^—Ni1—Tb2^v^	67.774 (19)	Ni4^xv^—Tb2—Ni2^xxiii^	48.48 (3)
Ni1^iii^—Ni1—Tb2^v^	112.228 (19)	Ni4^xix^—Tb2—Ni2^xxiii^	136.32 (4)
Ni1^iv^—Ni1—Tb2^v^	67.632 (19)	Ni4^xvii^—Tb2—Ni2^xxiii^	91.77 (4)
Ni5—Ni1—Tb2^v^	122.31 (3)	Ni3^xvii^—Tb2—Ni2^xxiii^	136.45 (4)
Tb1^v^—Ni1—Tb2^v^	69.68 (2)	Ni3^xv^—Tb2—Ni2^xxiii^	91.78 (4)
Tb1^vi^—Ni1—Tb2^v^	168.40 (3)	Ni3^xxii^—Tb2—Ni2^xxiii^	48.60 (3)
Tb1^vii^—Ni1—Tb2^v^	125.41 (2)	Ni4^xv^—Tb2—Ni2^xxiv^	91.77 (4)
Ni3—Ni1—Tb2^vi^	58.179 (19)	Ni4^xix^—Tb2—Ni2^xxiv^	48.48 (3)
Ni4—Ni1—Tb2^vi^	58.115 (19)	Ni4^xvii^—Tb2—Ni2^xxiv^	136.32 (4)
Ni1^i^—Ni1—Tb2^vi^	67.773 (19)	Ni3^xvii^—Tb2—Ni2^xxiv^	91.78 (4)
Ni1^ii^—Ni1—Tb2^vi^	112.370 (19)	Ni3^xv^—Tb2—Ni2^xxiv^	48.60 (3)
Ni1^iii^—Ni1—Tb2^vi^	67.632 (19)	Ni3^xxii^—Tb2—Ni2^xxiv^	136.45 (4)
Ni1^iv^—Ni1—Tb2^vi^	112.228 (19)	Ni2^xxiii^—Tb2—Ni2^xxiv^	87.891 (17)
Ni5—Ni1—Tb2^vi^	122.31 (3)	Ni4^xv^—Tb2—Ni2^viii^	136.32 (4)
Tb1^v^—Ni1—Tb2^vi^	168.40 (3)	Ni4^xix^—Tb2—Ni2^viii^	91.77 (4)
Tb1^vi^—Ni1—Tb2^vi^	69.68 (2)	Ni4^xvii^—Tb2—Ni2^viii^	48.48 (3)
Tb1^vii^—Ni1—Tb2^vi^	125.41 (2)	Ni3^xvii^—Tb2—Ni2^viii^	48.60 (3)
Tb2^v^—Ni1—Tb2^vi^	98.72 (3)	Ni3^xv^—Tb2—Ni2^viii^	136.45 (4)
Ni4^vii^—Ni2—Ni4	108.77 (8)	Ni3^xxii^—Tb2—Ni2^viii^	91.78 (4)
Ni4^vii^—Ni2—Ni3^vii^	71.21 (3)	Ni2^xxiii^—Tb2—Ni2^viii^	87.891 (17)
Ni4—Ni2—Ni3^vii^	179.98 (5)	Ni2^xxiv^—Tb2—Ni2^viii^	87.891 (17)
Ni4^vii^—Ni2—Ni3	179.98 (11)	Ni4^xv^—Tb2—Ni2^xxv^	91.77 (4)
Ni4—Ni2—Ni3	71.21 (3)	Ni4^xix^—Tb2—Ni2^xxv^	136.32 (4)
Ni3^vii^—Ni2—Ni3	108.81 (8)	Ni4^xvii^—Tb2—Ni2^xxv^	48.48 (3)
Ni4^vii^—Ni2—Ni2^iii^	59.71 (3)	Ni3^xvii^—Tb2—Ni2^xxv^	91.78 (4)
Ni4—Ni2—Ni2^iii^	59.72 (3)	Ni3^xv^—Tb2—Ni2^xxv^	136.45 (4)
Ni3^vii^—Ni2—Ni2^iii^	120.27 (3)	Ni3^xxii^—Tb2—Ni2^xxv^	48.60 (3)
Ni3—Ni2—Ni2^iii^	120.27 (3)	Ni2^xxiii^—Tb2—Ni2^xxv^	47.29 (6)
Ni4^vii^—Ni2—Ni2^iv^	59.71 (3)	Ni2^xxiv^—Tb2—Ni2^xxv^	106.52 (2)
Ni4—Ni2—Ni2^iv^	59.72 (3)	Ni2^viii^—Tb2—Ni2^xxv^	47.19 (6)
Ni3^vii^—Ni2—Ni2^iv^	120.27 (3)	Ni4^xv^—Tb2—Ni2^ix^	48.48 (3)
Ni3—Ni2—Ni2^iv^	120.27 (3)	Ni4^xix^—Tb2—Ni2^ix^	91.77 (4)
Ni2^iii^—Ni2—Ni2^iv^	60.0	Ni4^xvii^—Tb2—Ni2^ix^	136.32 (4)
Ni4^vii^—Ni2—Ni2^i^	120.29 (3)	Ni3^xvii^—Tb2—Ni2^ix^	136.45 (4)
Ni4—Ni2—Ni2^i^	120.28 (3)	Ni3^xv^—Tb2—Ni2^ix^	48.60 (3)
Ni3^vii^—Ni2—Ni2^i^	59.73 (3)	Ni3^xxii^—Tb2—Ni2^ix^	91.78 (4)
Ni3—Ni2—Ni2^i^	59.73 (3)	Ni2^xxiii^—Tb2—Ni2^ix^	47.19 (6)
Ni2^iii^—Ni2—Ni2^i^	120.0	Ni2^xxiv^—Tb2—Ni2^ix^	47.29 (6)
Ni2^iv^—Ni2—Ni2^i^	180.0	Ni2^viii^—Tb2—Ni2^ix^	106.52 (2)
Ni4^vii^—Ni2—Ni2^ii^	120.29 (3)	Ni2^xxv^—Tb2—Ni2^ix^	87.891 (17)
Ni4—Ni2—Ni2^ii^	120.28 (3)	Ni4^xv^—Tb2—Ni2^xxvi^	136.32 (4)
Ni3^vii^—Ni2—Ni2^ii^	59.73 (3)	Ni4^xix^—Tb2—Ni2^xxvi^	48.48 (3)
Ni3—Ni2—Ni2^ii^	59.73 (3)	Ni4^xvii^—Tb2—Ni2^xxvi^	91.77 (4)
Ni2^iii^—Ni2—Ni2^ii^	180.0	Ni3^xvii^—Tb2—Ni2^xxvi^	48.60 (3)
Ni2^iv^—Ni2—Ni2^ii^	120.001 (1)	Ni3^xv^—Tb2—Ni2^xxvi^	91.78 (4)
Ni2^i^—Ni2—Ni2^ii^	60.0	Ni3^xxii^—Tb2—Ni2^xxvi^	136.45 (4)
Ni4^vii^—Ni2—Tb2^viii^	60.919 (16)	Ni2^xxiii^—Tb2—Ni2^xxvi^	106.52 (2)
Ni4—Ni2—Tb2^viii^	119.12 (4)	Ni2^xxiv^—Tb2—Ni2^xxvi^	47.19 (6)
Ni3^vii^—Ni2—Tb2^viii^	60.876 (16)	Ni2^viii^—Tb2—Ni2^xxvi^	47.29 (6)
Ni3—Ni2—Tb2^viii^	119.09 (4)	Ni2^xxv^—Tb2—Ni2^xxvi^	87.891 (17)
Ni2^iii^—Ni2—Tb2^viii^	113.64 (3)	Ni2^ix^—Tb2—Ni2^xxvi^	87.891 (17)
Ni2^iv^—Ni2—Tb2^viii^	66.40 (3)	Ni1—Ni4—Ni1^iii^	61.06 (5)
Ni2^i^—Ni2—Tb2^viii^	113.60 (3)	Ni1—Ni4—Ni1^iv^	61.06 (5)
Ni2^ii^—Ni2—Tb2^viii^	66.36 (3)	Ni1^iii^—Ni4—Ni1^iv^	61.06 (5)
Ni4^vii^—Ni2—Tb2^v^	119.12 (4)	Ni1—Ni4—Ni2	108.47 (4)
Ni4—Ni2—Tb2^v^	60.918 (16)	Ni1^iii^—Ni4—Ni2	146.016 (17)
Ni3^vii^—Ni2—Tb2^v^	119.09 (4)	Ni1^iv^—Ni4—Ni2	146.016 (17)
Ni3—Ni2—Tb2^v^	60.876 (16)	Ni1—Ni4—Ni2^iv^	146.016 (17)
Ni2^iii^—Ni2—Tb2^v^	113.64 (3)	Ni1^iii^—Ni4—Ni2^iv^	146.015 (17)
Ni2^iv^—Ni2—Tb2^v^	66.40 (3)	Ni1^iv^—Ni4—Ni2^iv^	108.47 (4)
Ni2^i^—Ni2—Tb2^v^	113.60 (3)	Ni2—Ni4—Ni2^iv^	60.57 (6)
Ni2^ii^—Ni2—Tb2^v^	66.36 (3)	Ni1—Ni4—Ni2^iii^	146.015 (17)
Tb2^viii^—Ni2—Tb2^v^	73.48 (2)	Ni1^iii^—Ni4—Ni2^iii^	108.47 (4)
Ni4^vii^—Ni2—Tb2^vi^	119.12 (4)	Ni1^iv^—Ni4—Ni2^iii^	146.016 (17)
Ni4—Ni2—Tb2^vi^	60.919 (16)	Ni2—Ni4—Ni2^iii^	60.57 (6)
Ni3^vii^—Ni2—Tb2^vi^	119.09 (4)	Ni2^iv^—Ni4—Ni2^iii^	60.57 (6)
Ni3—Ni2—Tb2^vi^	60.876 (16)	Ni1—Ni4—Ni3^xxvii^	106.97 (4)
Ni2^iii^—Ni2—Tb2^vi^	66.40 (3)	Ni1^iii^—Ni4—Ni3^xxvii^	53.98 (4)
Ni2^iv^—Ni2—Tb2^vi^	113.64 (3)	Ni1^iv^—Ni4—Ni3^xxvii^	106.97 (4)
Ni2^i^—Ni2—Tb2^vi^	66.36 (3)	Ni2—Ni4—Ni3^xxvii^	107.02 (4)
Ni2^ii^—Ni2—Tb2^vi^	113.60 (3)	Ni2^iv^—Ni4—Ni3^xxvii^	107.02 (4)
Tb2^viii^—Ni2—Tb2^vi^	179.95 (6)	Ni2^iii^—Ni4—Ni3^xxvii^	54.49 (5)
Tb2^v^—Ni2—Tb2^vi^	106.52 (2)	Ni1—Ni4—Ni3	53.98 (4)
Ni4^vii^—Ni2—Tb2^ix^	60.919 (16)	Ni1^iii^—Ni4—Ni3	106.97 (4)
Ni4—Ni2—Tb2^ix^	119.12 (4)	Ni1^iv^—Ni4—Ni3	106.97 (4)
Ni3^vii^—Ni2—Tb2^ix^	60.877 (16)	Ni2—Ni4—Ni3	54.49 (5)
Ni3—Ni2—Tb2^ix^	119.09 (4)	Ni2^iv^—Ni4—Ni3	107.02 (4)
Ni2^iii^—Ni2—Tb2^ix^	66.40 (3)	Ni2^iii^—Ni4—Ni3	107.02 (4)
Ni2^iv^—Ni2—Tb2^ix^	113.64 (3)	Ni3^xxvii^—Ni4—Ni3	120.0
Ni2^i^—Ni2—Tb2^ix^	66.36 (3)	Ni1—Ni4—Ni3^xxviii^	106.97 (4)
Ni2^ii^—Ni2—Tb2^ix^	113.60 (3)	Ni1^iii^—Ni4—Ni3^xxviii^	106.97 (4)
Tb2^viii^—Ni2—Tb2^ix^	106.52 (2)	Ni1^iv^—Ni4—Ni3^xxviii^	53.98 (4)
Tb2^v^—Ni2—Tb2^ix^	179.95 (6)	Ni2—Ni4—Ni3^xxviii^	107.02 (4)
Tb2^vi^—Ni2—Tb2^ix^	73.48 (2)	Ni2^iv^—Ni4—Ni3^xxviii^	54.49 (5)
Ni1—Ni3—Ni1^i^	60.92 (5)	Ni2^iii^—Ni4—Ni3^xxviii^	107.02 (4)
Ni1—Ni3—Ni1^ii^	60.92 (5)	Ni3^xxvii^—Ni4—Ni3^xxviii^	120.0
Ni1^i^—Ni3—Ni1^ii^	60.92 (5)	Ni3—Ni4—Ni3^xxviii^	120.0
Ni1—Ni3—Ni2^i^	146.063 (17)	Ni1—Ni4—Tb2^v^	75.42 (2)
Ni1^i^—Ni3—Ni2^i^	108.58 (4)	Ni1^iii^—Ni4—Tb2^v^	128.83 (6)
Ni1^ii^—Ni3—Ni2^i^	146.064 (17)	Ni1^iv^—Ni4—Tb2^v^	75.42 (2)
Ni1—Ni3—Ni2^ii^	146.063 (17)	Ni2—Ni4—Tb2^v^	70.60 (2)
Ni1^i^—Ni3—Ni2^ii^	146.063 (17)	Ni2^iv^—Ni4—Tb2^v^	70.60 (2)
Ni1^ii^—Ni3—Ni2^ii^	108.58 (4)	Ni2^iii^—Ni4—Tb2^v^	122.70 (6)
Ni2^i^—Ni3—Ni2^ii^	60.54 (6)	Ni3^xxvii^—Ni4—Tb2^v^	177.19 (7)
Ni1—Ni3—Ni2	108.58 (4)	Ni3—Ni4—Tb2^v^	60.049 (2)
Ni1^i^—Ni3—Ni2	146.063 (17)	Ni3^xxviii^—Ni4—Tb2^v^	60.049 (2)
Ni1^ii^—Ni3—Ni2	146.063 (17)	Ni1—Ni4—Tb2^xxix^	128.83 (6)
Ni2^i^—Ni3—Ni2	60.54 (6)	Ni1^iii^—Ni4—Tb2^xxix^	75.42 (2)
Ni2^ii^—Ni3—Ni2	60.54 (6)	Ni1^iv^—Ni4—Tb2^xxix^	75.42 (2)
Ni1—Ni3—Ni4^x^	107.11 (4)	Ni2—Ni4—Tb2^xxix^	122.70 (6)
Ni1^i^—Ni3—Ni4^x^	54.28 (4)	Ni2^iv^—Ni4—Tb2^xxix^	70.60 (2)
Ni1^ii^—Ni3—Ni4^x^	107.11 (4)	Ni2^iii^—Ni4—Tb2^xxix^	70.60 (2)
Ni2^i^—Ni3—Ni4^x^	54.30 (5)	Ni3^xxvii^—Ni4—Tb2^xxix^	60.048 (2)
Ni2^ii^—Ni3—Ni4^x^	106.83 (4)	Ni3—Ni4—Tb2^xxix^	177.19 (7)
Ni2—Ni3—Ni4^x^	106.83 (4)	Ni3^xxviii^—Ni4—Tb2^xxix^	60.049 (2)
Ni1—Ni3—Ni4^xi^	107.11 (4)	Tb2^v^—Ni4—Tb2^xxix^	119.745 (6)
Ni1^i^—Ni3—Ni4^xi^	107.11 (4)	Ni1—Ni4—Tb2^vi^	75.42 (2)
Ni1^ii^—Ni3—Ni4^xi^	54.28 (4)	Ni1^iii^—Ni4—Tb2^vi^	75.42 (2)
Ni2^i^—Ni3—Ni4^xi^	106.83 (4)	Ni1^iv^—Ni4—Tb2^vi^	128.83 (6)
Ni2^ii^—Ni3—Ni4^xi^	54.30 (5)	Ni2—Ni4—Tb2^vi^	70.60 (2)
Ni2—Ni3—Ni4^xi^	106.83 (4)	Ni2^iv^—Ni4—Tb2^vi^	122.70 (6)
Ni4^x^—Ni3—Ni4^xi^	120.0	Ni2^iii^—Ni4—Tb2^vi^	70.60 (2)
Ni1—Ni3—Ni4	54.28 (4)	Ni3^xxvii^—Ni4—Tb2^vi^	60.049 (2)
Ni1^i^—Ni3—Ni4	107.11 (4)	Ni3—Ni4—Tb2^vi^	60.049 (2)
Ni1^ii^—Ni3—Ni4	107.11 (4)	Ni3^xxviii^—Ni4—Tb2^vi^	177.19 (7)
Ni2^i^—Ni3—Ni4	106.83 (4)	Tb2^v^—Ni4—Tb2^vi^	119.743 (6)
Ni2^ii^—Ni3—Ni4	106.83 (4)	Tb2^xxix^—Ni4—Tb2^vi^	119.743 (6)
Ni2—Ni3—Ni4	54.30 (5)	Ni1^xxx^—Ni5—Ni1^xxxi^	59.12 (4)
Ni4^x^—Ni3—Ni4	120.0	Ni1^xxx^—Ni5—Ni1^iv^	120.88 (4)
Ni4^xi^—Ni3—Ni4	120.0	Ni1^xxxi^—Ni5—Ni1^iv^	180.00 (2)
Ni1—Ni3—Tb2^v^	75.55 (2)	Ni1^xxx^—Ni5—Ni1^iii^	120.88 (4)
Ni1^i^—Ni3—Tb2^v^	128.85 (6)	Ni1^xxxi^—Ni5—Ni1^iii^	120.88 (4)
Ni1^ii^—Ni3—Tb2^v^	75.55 (2)	Ni1^iv^—Ni5—Ni1^iii^	59.12 (4)
Ni2^i^—Ni3—Tb2^v^	122.57 (6)	Ni1^xxx^—Ni5—Ni1^xxxii^	59.12 (4)
Ni2^ii^—Ni3—Tb2^v^	70.52 (2)	Ni1^xxxi^—Ni5—Ni1^xxxii^	59.12 (4)
Ni2—Ni3—Tb2^v^	70.52 (2)	Ni1^iv^—Ni5—Ni1^xxxii^	120.88 (4)
Ni4^x^—Ni3—Tb2^v^	176.87 (7)	Ni1^iii^—Ni5—Ni1^xxxii^	180.00 (2)
Ni4^xi^—Ni3—Tb2^v^	60.039 (2)	Ni1^xxx^—Ni5—Ni1	180.0
Ni4—Ni3—Tb2^v^	60.040 (2)	Ni1^xxxi^—Ni5—Ni1	120.88 (4)
Ni1—Ni3—Tb2^xii^	128.85 (6)	Ni1^iv^—Ni5—Ni1	59.12 (4)
Ni1^i^—Ni3—Tb2^xii^	75.55 (2)	Ni1^iii^—Ni5—Ni1	59.12 (4)
Ni1^ii^—Ni3—Tb2^xii^	75.55 (2)	Ni1^xxxii^—Ni5—Ni1	120.88 (4)
Ni2^i^—Ni3—Tb2^xii^	70.52 (2)	Ni1^xxx^—Ni5—Tb1^xxxiii^	61.922 (12)
Ni2^ii^—Ni3—Tb2^xii^	70.52 (2)	Ni1^xxxi^—Ni5—Tb1^xxxiii^	61.922 (12)
Ni2—Ni3—Tb2^xii^	122.57 (6)	Ni1^iv^—Ni5—Tb1^xxxiii^	118.078 (12)
Ni4^x^—Ni3—Tb2^xii^	60.039 (2)	Ni1^iii^—Ni5—Tb1^xxxiii^	68.92 (3)
Ni4^xi^—Ni3—Tb2^xii^	60.040 (2)	Ni1^xxxii^—Ni5—Tb1^xxxiii^	111.08 (3)
Ni4—Ni3—Tb2^xii^	176.87 (7)	Ni1—Ni5—Tb1^xxxiii^	118.078 (12)
Tb2^v^—Ni3—Tb2^xii^	119.725 (6)	Ni1^xxx^—Ni5—Tb1^v^	118.078 (12)
Ni1—Ni3—Tb2^vi^	75.55 (2)	Ni1^xxxi^—Ni5—Tb1^v^	118.078 (12)
Ni1^i^—Ni3—Tb2^vi^	75.55 (2)	Ni1^iv^—Ni5—Tb1^v^	61.922 (12)
Ni1^ii^—Ni3—Tb2^vi^	128.85 (6)	Ni1^iii^—Ni5—Tb1^v^	111.08 (3)
Ni2^i^—Ni3—Tb2^vi^	70.52 (2)	Ni1^xxxii^—Ni5—Tb1^v^	68.92 (3)
Ni2^ii^—Ni3—Tb2^vi^	122.57 (6)	Ni1—Ni5—Tb1^v^	61.922 (12)
Ni2—Ni3—Tb2^vi^	70.52 (2)	Tb1^xxxiii^—Ni5—Tb1^v^	180.00 (2)
Ni4^x^—Ni3—Tb2^vi^	60.039 (2)	Ni1^xxx^—Ni5—Tb1^vii^	111.08 (3)
Ni4^xi^—Ni3—Tb2^vi^	176.87 (7)	Ni1^xxxi^—Ni5—Tb1^vii^	61.922 (12)
Ni4—Ni3—Tb2^vi^	60.040 (2)	Ni1^iv^—Ni5—Tb1^vii^	118.078 (12)
Tb2^v^—Ni3—Tb2^vi^	119.725 (6)	Ni1^iii^—Ni5—Tb1^vii^	118.078 (12)
Tb2^xii^—Ni3—Tb2^vi^	119.724 (6)	Ni1^xxxii^—Ni5—Tb1^vii^	61.922 (12)
Ni1^xiii^—Tb1—Ni1^xiv^	51.68 (5)	Ni1—Ni5—Tb1^vii^	68.92 (3)
Ni1^xiii^—Tb1—Ni1^xv^	98.43 (2)	Tb1^xxxiii^—Ni5—Tb1^vii^	114.616 (9)
Ni1^xiv^—Tb1—Ni1^xv^	52.01 (5)	Tb1^v^—Ni5—Tb1^vii^	65.384 (9)
Ni1^xiii^—Tb1—Ni1^xvi^	121.92 (4)	Ni1^xxx^—Ni5—Tb1^xxix^	68.92 (3)
Ni1^xiv^—Tb1—Ni1^xvi^	98.43 (2)	Ni1^xxxi^—Ni5—Tb1^xxix^	118.078 (12)
Ni1^xv^—Tb1—Ni1^xvi^	51.68 (5)	Ni1^iv^—Ni5—Tb1^xxix^	61.922 (12)
Ni1^xiii^—Tb1—Ni1^xvii^	52.01 (5)	Ni1^iii^—Ni5—Tb1^xxix^	61.922 (12)
Ni1^xiv^—Tb1—Ni1^xvii^	98.43 (2)	Ni1^xxxii^—Ni5—Tb1^xxix^	118.078 (12)
Ni1^xv^—Tb1—Ni1^xvii^	121.92 (4)	Ni1—Ni5—Tb1^xxix^	111.08 (3)
Ni1^xvi^—Tb1—Ni1^xvii^	98.43 (2)	Tb1^xxxiii^—Ni5—Tb1^xxix^	65.384 (9)
Ni1^xiii^—Tb1—Ni1^xviii^	98.43 (2)	Tb1^v^—Ni5—Tb1^xxix^	114.616 (9)
Ni1^xiv^—Tb1—Ni1^xviii^	121.92 (4)	Tb1^vii^—Ni5—Tb1^xxix^	180.00 (2)
Ni1^xv^—Tb1—Ni1^xviii^	98.43 (2)	Ni1^xxx^—Ni5—Tb1^vi^	118.078 (12)
Ni1^xvi^—Tb1—Ni1^xviii^	52.01 (5)	Ni1^xxxi^—Ni5—Tb1^vi^	68.92 (3)
Ni1^xvii^—Tb1—Ni1^xviii^	51.68 (5)	Ni1^iv^—Ni5—Tb1^vi^	111.08 (3)
Ni1^xiii^—Tb1—Ni5^xv^	96.49 (2)	Ni1^iii^—Ni5—Tb1^vi^	61.922 (12)
Ni1^xiv^—Tb1—Ni5^xv^	51.64 (2)	Ni1^xxxii^—Ni5—Tb1^vi^	118.078 (12)
Ni1^xv^—Tb1—Ni5^xv^	51.64 (2)	Ni1—Ni5—Tb1^vi^	61.922 (12)
Ni1^xvi^—Tb1—Ni5^xv^	96.49 (2)	Tb1^xxxiii^—Ni5—Tb1^vi^	65.385 (9)
Ni1^xvii^—Tb1—Ni5^xv^	148.32 (2)	Tb1^v^—Ni5—Tb1^vi^	114.615 (9)
Ni1^xviii^—Tb1—Ni5^xv^	148.32 (2)	Tb1^vii^—Ni5—Tb1^vi^	65.385 (9)
Ni1^xiii^—Tb1—Ni5^xix^	148.32 (2)	Tb1^xxix^—Ni5—Tb1^vi^	114.615 (9)
Ni1^xiv^—Tb1—Ni5^xix^	148.32 (2)	Ni1^xxx^—Ni5—Tb1^xxxiv^	61.922 (12)
Ni1^xv^—Tb1—Ni5^xix^	96.49 (2)	Ni1^xxxi^—Ni5—Tb1^xxxiv^	111.08 (3)
Ni1^xvi^—Tb1—Ni5^xix^	51.64 (2)	Ni1^iv^—Ni5—Tb1^xxxiv^	68.92 (3)
Ni1^xvii^—Tb1—Ni5^xix^	96.49 (2)	Ni1^iii^—Ni5—Tb1^xxxiv^	118.078 (12)
Ni1^xviii^—Tb1—Ni5^xix^	51.64 (2)	Ni1^xxxii^—Ni5—Tb1^xxxiv^	61.922 (12)
Ni5^xv^—Tb1—Ni5^xix^	114.616 (9)	Ni1—Ni5—Tb1^xxxiv^	118.078 (12)
Ni1^xiii^—Tb1—Ni5^xvii^	51.64 (2)	Tb1^xxxiii^—Ni5—Tb1^xxxiv^	114.615 (9)
Ni1^xiv^—Tb1—Ni5^xvii^	96.49 (2)	Tb1^v^—Ni5—Tb1^xxxiv^	65.385 (9)
Ni1^xv^—Tb1—Ni5^xvii^	148.32 (2)	Tb1^vii^—Ni5—Tb1^xxxiv^	114.615 (9)
Ni1^xvi^—Tb1—Ni5^xvii^	148.32 (2)	Tb1^xxix^—Ni5—Tb1^xxxiv^	65.385 (9)
Ni1^xvii^—Tb1—Ni5^xvii^	51.64 (2)	Tb1^vi^—Ni5—Tb1^xxxiv^	180.00 (2)

Symmetry codes: (i) −*x*+*y*, −*x*+1, *z*; (ii) −*y*+1, *x*−*y*+1, *z*; (iii) −*y*, *x*−*y*, *z*; (iv) −*x*+*y*, −*x*, *z*; (v) −*x*+1, −*y*+1, *z*−1/2; (vi) −*x*, −*y*+1, *z*−1/2; (vii) *x*, *y*, −*z*+1/2; (viii) −*x*+1, −*y*+1, −*z*+1; (ix) −*x*, −*y*+1, −*z*+1; (x) *x*, *y*+1, *z*; (xi) *x*+1, *y*+1, *z*; (xii) −*x*+1, −*y*+2, *z*−1/2; (xiii) *x*−*y*+1, *x*+1, *z*+1/2; (xiv) *y*, −*x*+*y*+1, *z*+1/2; (xv) −*x*, −*y*+1, *z*+1/2; (xvi) *x*−*y*, *x*, *z*+1/2; (xvii) −*x*+1, −*y*+1, *z*+1/2; (xviii) *y*, −*x*+*y*, *z*+1/2; (xix) −*x*, −*y*, *z*+1/2; (xx) −*x*+*y*, −*x*+1, −*z*+1/2; (xxi) −*y*+1, *x*−*y*+1, −*z*+1/2; (xxii) −*x*+1, −*y*+2, *z*+1/2; (xxiii) *y*, −*x*+*y*+1, −*z*+1; (xxiv) *x*−*y*, *x*, −*z*+1; (xxv) *x*−*y*+1, *x*+1, −*z*+1; (xxvi) *y*, −*x*+*y*, −*z*+1; (xxvii) *x*−1, *y*−1, *z*; (xxviii) *x*, *y*−1, *z*; (xxix) −*x*, −*y*, *z*−1/2; (xxx) −*x*, −*y*, −*z*; (xxxi) *x*−*y*, *x*, −*z*; (xxxii) *y*, −*x*+*y*, −*z*; (xxxiii) *x*−1, *y*−1, −*z*+1/2; (xxxiv) *x*, *y*−1, −*z*+1/2.
